# Integrative cognitive neuropsychological program emphasizing brain response to enhance inhibitory control among substance abusers at the alternative rehabilitation communities in Eastern Thailand

**DOI:** 10.3389/fpsyt.2025.1531443

**Published:** 2025-08-20

**Authors:** Juthamas Haenjohn, Warakorn Supwirapakorn, Jatuporn Namyen

**Affiliations:** Department of Research and Applied Psychology, Faculty of Education, Burapha University, Chonburi, Thailand

**Keywords:** inhibitory control, integrative cognitive neuropsychological program, brain response, substance abusers, cognitive rehabilitation, alternative rehabilitation communities

## Abstract

**Introduction:**

Inhibitory control (IC) deficit among substance abusers is a potential consequence of detrimental drug use and can also serve as a risk factor for drug-seeking behaviors, compromising substance abuse treatment and leading to drug relapse. This study examined the efficacy of an integrative cognitive neuropsychological program emphasizing brain response to enhance inhibitory control (ICNIC intervention program) among substance abusers.

**Methods:**

A total of 30 substance abusers were recruited and randomly assigned to either an ICNIC training group and a non-training control group. The ICNIC training group participated in a 12-session intervention program and a regular rehabilitative program at alternative treatment centers. The control group did not receive ICNIC training, but participated in only the regular rehabilitative program. IC was assessed using self-report measures and a cognitive performance task. Data were collected at three points: before ICNIC training, after ICNIC training, and at a 3-week follow-up ICNIC training. Statistical analyses were implemented.

**Results:**

The primary findings showed that substance abusers exhibited greater self-reported self-efficacy of behavioral IC after the ICNIC training, as assessed by the self-report measure, Behavioral Inhibitory Control Inventory – Substance Use (BICI-SU). There were no improvements in the ‘Go’ or ‘Stop’ trial accuracy (neither within-subject improvement nor between-group difference at the post-training or follow-up periods), and SSRT reaction time (neither pre- to post-training within-subject improvement nor between-group difference at post-training or follow-up periods), as measured by Stop Signal Substance Task (SSST). Moreover, the lack of improved accuracy coincided with slower responses to ‘Go’ stimuli in the ICNIC training group as compared to the non-training control group. Thus, the ICNIC training improved self-reported self-efficacy in IC and, therefore, may be associated with an improved and cautious response strategies for IC paradigms, resulting in slower response times. However, these strategies did not translate to improved response accuracy during the SSRT.

**Conclusion:**

The implementation of an ICNIC intervention program as a supplementary cognitive rehabilitation approach could potentially enhance self-efficacy of behavioral IC and improve response strategies among substance abusers. With further investigation, the program may be expected to contribute to an increase in cognitive control and promote behavioral changes that lead to positive therapeutic outcomes.

## 1 Introduction

### 1.1 Drug addiction and drug-induced cognitive changes

Drug addiction has been increasing worldwide, with an approximated 296 million drug users in 2021, and the number continues to grow. The World Drug Report (2023) indicated that an estimated 1 in 17 people had used drugs in the past year. Cannabis was the most commonly used drug worldwide with 219 million users, followed by opioids (60 million users), amphetamines (36 million), cocaine (22 million), and ecstasy-type substances (20 million) (1).

In Thailand, the rise of drug use is a major public health concern with the growing drug use and treatment admissions among illicit drug users, including crystal methamphetamine, methamphetamine tablets, ecstasy, ketamine, cannabis herb, heroin, kratom, and cocaine (2). Furthermore, the widespread use of drug cocktails, known as “happy water,” has been raising the alarm for nighttime partygoers. Happy water is a psychoactive beverage that can be produced by mixing synthetic drugs into a sweetened drink, typically including methamphetamine, ecstasy, ketamine, caffeine, diazepam, and tramadol all in one (3).

Evidence shows that amphetamine-type stimulants (ATS) are the most commonly used drugs in Thailand. ATS consist of synthetic drugs such as amphetamine, methamphetamine, ephedrine, pseudoephedrine, and ecstasy (MDMA) along with its derivatives (4). With its psychoactive properties, ATS exert potent neurotoxic and neurocognitive effects upon the brain (5). Both human and experimental animal studies have shown the neurotoxic and neuroinflammatory effects of amphetamine-related drugs that can lead to neuronal cell death, such as oxidative stress, excitotoxicity, reactive gliosis, cell apoptosis, and DNA damage (6, 7), subsequently resulting in cognitive deficits and neuropsychiatric symptoms among abusers. As a result, several previous findings have indicated that chronic methamphetamine use can result in cognitive impairment, including memory loss, attention deficit, impulsivity, executive dysfunction, learning impairment, and poor decision making (8). Additionally, methamphetamine users often show manifestations of psychiatric symptoms such as dependence, agitation, anxiety, hallucinations, paranoia, aggression, and psychosis (6). Previous study among Thai methamphetamine abusers has shown that these users with psychiatric symptoms exhibited even higher cognitive impairment than those without psychiatric symptoms, such as psychosis (9).

Chronic drug use induces significant changes in brain regions associated with cognitive functions, leading to cognitive deficits (10). Studies using functional and structural neuroimaging techniques in methamphetamine abusers have demonstrated that chronic drug use can cause damage to numerous brain regions in the frontal, parietal, and temporal lobes. Some of these regions play a significant role in cognitive function, addiction, and emotional regulation. For example, the prefrontal cortex (PFC) is an essential brain region for executive functions. The striatum functions in reward processes and addiction. The limbic system plays a role in emotional regulation, impulsivity, and aggressive behavior among abusers (11). Thus, brain damage associated with chronic psychoactive drug use results in cognitive deficits and neuropsychiatric symptoms, which subsequently has a detrimental impact on addiction treatment and drug use relapse.

As evaluated by the standardized cognitive screening tool, Montreal Cognitive Assessment (MoCA), approximately 70% of chronic methamphetamine abusers exhibit cognitive impairments (12). Previous meta-analysis has shown predominant deficits in impulsivity, reward processing, and social cognition and moderate deficits in executive functions, attention, working memory, visual memory, verbal learning and memory, and language/verbal fluency (13). Impairment of these higher brain cognitive processes is the adverse consequence of illicit drug usage on PFC, which is brain region responsible for executive functions (14).

Individuals with different types of substance use disorders (SUDs), including alcohol, cannabis, opioids, and stimulants, also exhibit cognitive impairments (15) with deficits in several domains, such as executive functions (e.g., shifting, inhibition, and working memory), attention (e.g., selective attention, attentional biases towards drug-related stimuli), memory (e.g., episodic memory), visuospatial abilities, and reward-based decision-making (15, 16).

The most common cause of cognitive deficits in substance abusers, however, is linked to prolonged drug use. For example, acute methamphetamine users exhibit an enhanced cognitive performance in terms of visuospatial perception, attention, and inhibition while, on the contrary, long-term use of methamphetamines is often linked to cognitive impairments (17).

Inhibitory control means the ability to self-regulate, suppress distractions to maintain focus, and appropriately manage thoughts. Inhibitory control signifies the human capability to think before acting, resist temptations, and overcome unexpected obstacles or problems (18, 19). Inhibitory control has been conceptualized as one of core components of executive functions (20). Diamond’s conceptual framework of executive functions is comprised of three core components: inhibitory control, working memory, and cognitive flexibility. Diamond has conceptualized inhibitory control as the capacity to control one’s behavior to overcome an instinctive reaction, such as the drive to use drugs, and instead carry on with tasks to achieve higher order goals (18).

Individuals with SUDs exhibit particular cognitive deficits in executive functions. The association between poor inhibitory control and an increased risk of substance abuse in adolescents has also been emphasized (21). Indeed, adolescence is the period of an on-going development of inhibitory control (22). Neuroimaging studies have implicated the brain neural networks and the contribution of impaired inhibitory control as a risk factor on developing substance abuse (23–25). In parallel, prolonged drug use can lead to deficits in inhibitory control among many drug users, subsequently leading to cravings and relapse following periods of abstinence (26, 27). Previous studies demonstrated that dysfunction of frontal brain regions involved with inhibitory control may lead to the loss of control of the limbic system. Thus, when experiencing drug cravings or withdrawal symptoms, the brain’s higher cognitive processes are unable to effectively inhibit the drive of drug seeking behaviors (28, 29).

### 1.2 Cognitive rehabilitation in drug addiction context

Drug-induced cognitive deficits in substance abusers can be restored using two approaches: computerized cognitive training and cognitive rehabilitation (16). Cognitive training contributes to an improvement in cognitive function in individuals with SUDs; additionally, it can be beneficial for addiction treatment by ameliorating cognitive deficits, alleviating addiction symptoms, and reducing the risk of relapse (30). Many types of cognitive training are implemented using computer programs and videogame-based training, targeting cognitive domains, such as memory, executive functions, abstract reasoning, problem solving, and processing speed (30). Previous studies have shown the efficacy of numerous computerized cognitive training programs and software on the enhancement of cognitive domains in frontal brain regions, including sustained attention, verbal memory, problem-solving, decision-making, cognitive flexibility (31), impulsivity, impulsive control, self-regulation (32, 33), and processing speed (34) among individuals with SUDs.

Meanwhile, cognitive rehabilitation or remediation emphasizes meta-cognitive training and strategy learning by focusing on goal-directed behaviors and decision making in real-world tasks. Top-down goal-driven behaviors require greater complexity and entropy to adapt cognitive strategies to both daily context and future goals. Therefore, cognitive rehabilitation approaches are considered to be more suitable (16). Cognitive rehabilitation or remediation techniques can be utilized to normalize the aberrant activities of brain reward systems and strengthen inhibitory control network of the brain. These techniques include standard cognitive behavioral therapy (CBT), cognitive inhibition of craving, motivational interventions, emotion regulation, mindfulness, and neurofeedback training on addiction (35).

In addition, the third wave of CBT, particularly Acceptance and Commitment Therapy (ACT) have been implemented in treating SUDs (36). ACT has been shown to effectively treat a wide variety of SUDs, including polydrug use (37), smoking (38), opioids (39), and amphetamines (40). ACT increases psychological flexibility, subsequently reducing aberrant psychological symptoms, such as shame, depression, and anxiety, among individuals with SUDs (37, 41–43). Other studies have shown that ACT can effectively reduce impulsivity and its components, including cognitive impulsivity, non-motor impulsivity, and non-planning impulsivity (44). Furthermore, it also shows a trend toward positive changes in executive functions, including inhibitory control, task monitoring, and emotional control (45). Thus, top-down executive control training with cognitive rehabilitation might be beneficial in reducing drug seeking behaviors and relapse.

### 1.3 The present study

**Figure 1:**
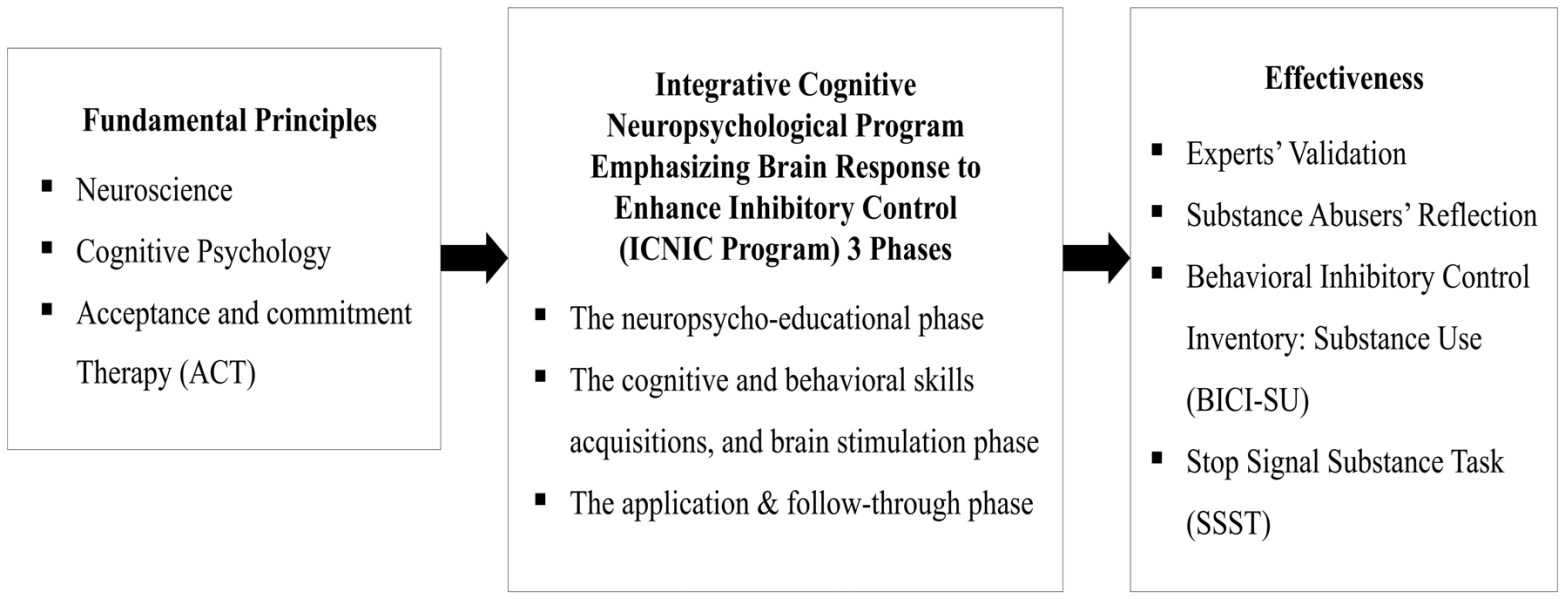
Conceptual framework of this research.

From the aforementioned literature reviews, there are, however, no established cognitive and behavioral interventions that directly and effectively improve inhibitory control among substance abusers in Thailand. This research study therefore aimed to examine the effectiveness of an integrative cognitive neuropsychological program emphasizing brain response to enhance inhibitory control (ICNIC intervention program) among substance abusers in Thailand. Fundamental principles of neuroscience, cognitive psychology, and Acceptance and Commitment Therapy (ACT) were applied to the development of an ICNIC intervention program. The effectiveness of this program was evaluated by both quantitative and qualitative approaches. The conceptual framework of this research study is demonstrated in **Figure 1**.

## 2 Materials and methods

### 2.1 Participants and procedure

This research study was designed as a pilot randomized controlled trial using both quantitative and qualitative research methods to investigate the efficacy of a cognitive rehabilitation program, known as the ‘integrative cognitive neuropsychological program emphasizing brain response to enhance inhibitory control’ (ICNIC intervention program), upon substance abusers who were outpatients at alternative drug treatment centers in several Eastern regions of Thailand (see **Figure 1** for research conceptual framework).

Thirty substance abusers (N=30) were selected for this experimental design with the sample size based on methods of F. N. Kerlinger and W. Wiersma (46, 47). These subjects were being treated at alternative drug treatment centers, which were selected using cluster random sampling. Participant inclusion criteria included a) age between 18-60 years old with at least 6 months of admission to the rehabilitation centers, b) being able to communicate in Thai fluently, c) absence of brain trauma and psychiatric illnesses based on medical history, d) right-handed as assessed by the Edinburgh Handedness Inventory (48), e) obtaining a low to moderate score in both our self-report measure and cognitive performance task, f) no history of opioid use that may affect inhibitory control, and g) voluntary participation.

Incomplete participation and the use of medications that might affect inhibitory control were considered as exclusion criteria in this study. Recruited participants were treated in accordance with strict ethical guidelines being submitted for ethical review and approval from the Human Research Ethics Committee of Burapha University (approval number IRB1-116/2023).

Simple random sampling was used to assign these participants into either the experimental group (N=15) and control group (N=15). The experimental group consisted of substance abusers who participated the ICNIC training intervention program and regular rehabilitative programs at the alternative treatment centers, while the control group included substance abusers who continued to participate in only regular rehabilitative programs at the alternative treatment centers. Quantitative data were collected using a demographic questionnaire, a self-report measure, the Behavioral Inhibitory Control Inventory-Substance Use (BICI-SU), and a cognitive performance task, the Stop Signal Substance Task (SSST). Participant feedback and evaluations of the ICNIC intervention program were collected as both quantitative and qualitative data.

### 2.2 Self-report measure

#### 2.2.1 Behavioral inhibitory control inventory - substance use

Behavioral Inhibitory Control Inventory - Substance Use (BICI-SU) is a self-report measure developed by the present researcher based on Adele Diamond's conceptual framework of executive functions (18, 49). BICI-SU is constructed to measure behaviors associated with inhibitory control in the context of drug use during the previous 6 months. It contains 45 items with two main components and four subcomponents: interference control (cognitive inhibition and selective attention) and response inhibition (self-control and discipline) with a 10-minute time completion limit. The items are scored on a six-point Likert scale (1=*never*, 2=*rarely*, 3=*sometimes*, 4=*fairly often*, 5=often, and 6=*always*). The Cronbach’s alpha coefficient was 0.97, and item discrimination values ranged between 0.26 - 0.77. The coefficients of variation ranged from 20.37 to 25.05.

### 2.3 Cognitive performance task

#### 2.3.1 Stop signal substance task

Stop Signal Substance Task (SSST) is a computer-based task developed to measure the cognitive performance of inhibitory control with the integration of different experimental paradigms, emphasizing the inhibition of substance use (50–56). It consists of Go trials (drug images paired with white circles around white arrows, 50% left arrows and 50% right arrows) and Stop trials (drug images paired with red circles around white arrows), with a total of 250 trials (200 Go trials and 50 Stop trials, in an 80:20 ratio). Two types of stop signals were implemented: visual and auditory. For the auditory signal, drug images were paired with red circles around white arrows that appear simultaneously with a beep sound. The Stop Signal Delay (SSD) is 250 ± 50 milliseconds. The scoring criteria for SSST were based on the Stop Signal Reaction Time (SSRT), and were calculated by subtracting the Stop Signal Delay (SSD) from the correct Go Reaction Time (Go RT).

**Figure 2:**
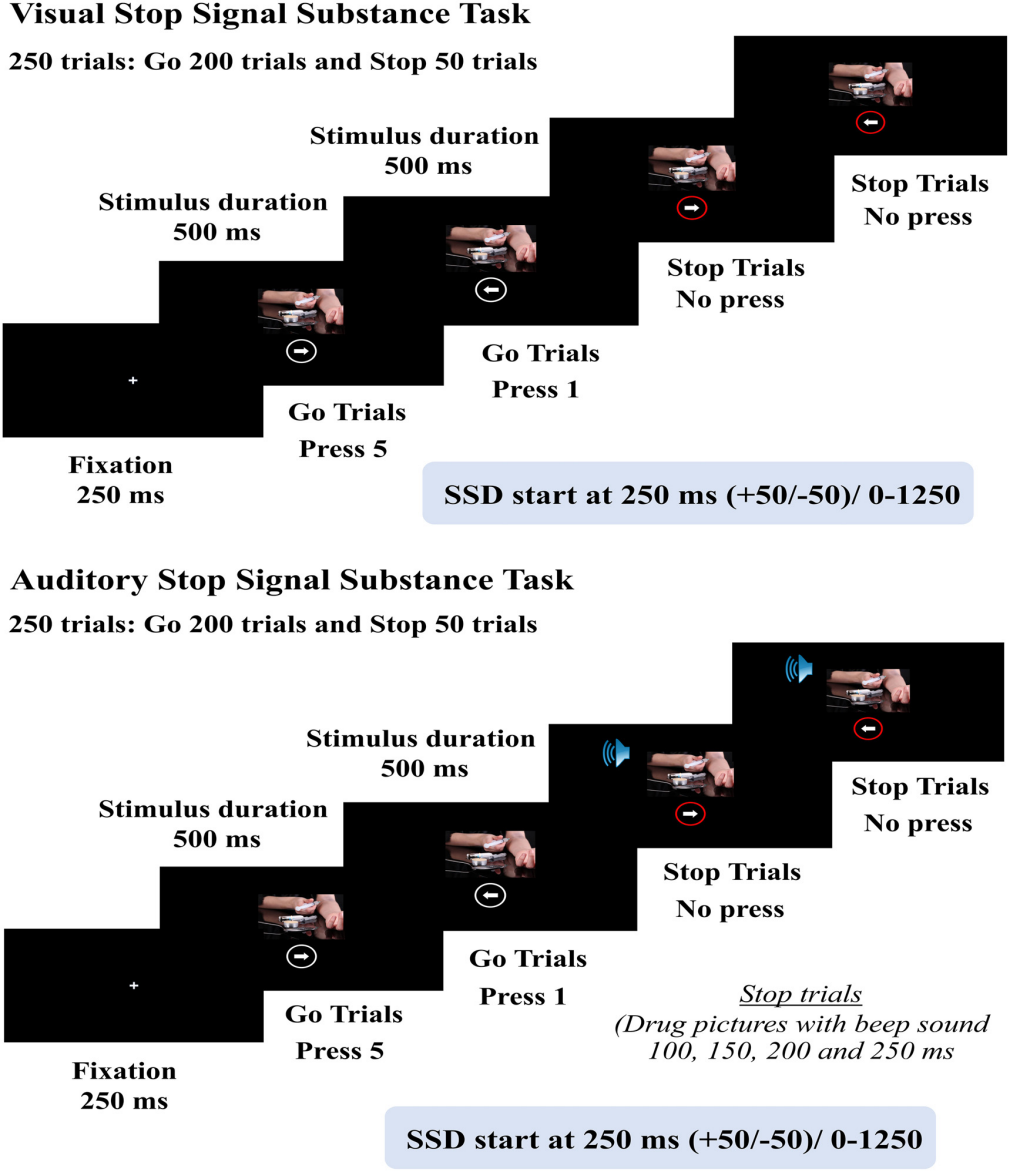
Schematic representation of Stop Signal Substance Task (SSST), both visual and auditory tasks.

Impairment of inhibitory control could be demonstrated using the longer SSRT and lower accuracy scores of Go trials. The test-retest reliability coefficients of the Visual SSST were as follows: %Go ACC =0.73, %St ACC = 0.78, SSD = 0.81, GoRT = 0.48, and SSRT = 0.65. For the auditory SSST, the test-retest reliability coefficients were: %Go ACC = 0.85, GoRT = 0.75, %St ACC = 0.81, SSD = 0.52, and SSRT = 0.53 (**Figure 2**).

### 2.4 Cognitive rehabilitation program

#### 2.4.1 Integrative cognitive neuropsychological program emphasizing brain response to enhance inhibitory control (ICNIC intervention program)

The ICNIC intervention program was developed using a multidisciplinary approach that integrates principles from neuroscience, cognitive psychology, and Acceptance and Commitment Therapy (ACT) to establish values and commitment in strengthening inhibitory control on substance use.

The concept of inhibitory control is based on Diamond’s conceptual framework (18, 49). Additionally, the ICNIC intervention program implemented integrated knowledge and learnable skills promoting the inhibition of drug use in rehabilitation centers. It includes skill training and learning activities related to inhibitory control, consisting of 12 sessions (duration: 50 minutes per session x 2 weeks). Nine sessions were performed in a marathon format within first two days (Day 1 and Day 2) and a brief 50-minute session on Day 5, Day 10, and Day 14 (3 sessions). The program was evaluated by 5 qualified experts in neuroscience, psychiatry, psychiatric nursing, and psychometrics to confirm its suitability and consistency with the program’s objectives, applied theoretical concepts, and targeted population.

The ICNIC intervention program is divided into 3 phases. Phase 1: the neuropsycho-educational phase, which consisted of 3 sessions (1. building rapport, 2. learning for brain plasticity, and 3. brain inhibitory control). Phase 2: cognitive and behavioral skill acquisitions, brain stimulation phase of which consisted of 6 sessions (4. values and goals, 5. parts of life, 6. observation without judgment, 7. inhibiting craving, 8. selective attention, and 9. the world of reality). Phase 3: The application and follow-through phase consisted of 3 sessions (10. checking in, 11. quickness and flexibility, and 12. integration and termination).

### 2.5 Statistical analysis

Descriptive statistics were performed using means, standard deviations, frequencies, and percentages. A repeated measures analysis of variance (ANOVA) with one between-group variable and one within-group variable was employed to compare scores of the BICI-SU and SSST during 1) the pre-ICNIC training period, 2) the post-ICNIC training period, and 3) a 3-week follow-up ICNIC training period, in which comparisons were made between an ICNIC training group and a non-training control group. The pairwise comparisons were performed using the Bonferroni method. The qualitative data were analyzed using thematic analysis. Data analyses were conducted using a specialized statistical program.

## 3 Results

### 3.1 Demographic and clinical characteristics of substance abusers

A total of 30 substance abusers were recruited from alternative drug rehabilitation centers and randomly assigned into: 1) an ICNIC training group (N=15), who participated in the ICNIC training program along with a regular rehabilitative program, and 2) non-training control group (N=15), which participated in only a regular rehabilitative program.

Demographic data revealed that the majority of substance abusers in both the ICNIC training group (53.33%) and control group (46.67%), engaged in polydrug use. The second most frequently type of substance use was ice (30.00%), followed by amphetamines (13.33%), and combined amphetamines-ice (3.33%), respectively. A majority of subjects in both groups exhibited more than ten years of drug use (66.67%), followed by two to five years (16.67%), six to ten years (10.00%), and less than two years (6.67%). Most participants were admitted for rehabilitation durations between less than one year and two years, with no reported physical illnesses. Although one substance abuser in the control group reported experiencing depression symptoms no such clinical diagnosis had been made (**Table 1**).

**Table 1:** Demographic and clinical characteristics of substance abusers (N=30).

Demographical characteristics	ICNIC group (N=15)		Control group (N=15)		Total participants (N=30)	
	N	%	N	%	N	%
Educational level						
Below undergraduate levels (≤ 12 years)	13	86.63	13	86.63	26	86.67
Undergraduate levels (> 12 years)	2	13.33	2	13.33	4	13.33
Marital status						
Single	13	86.63	13	86.63	26	86.67
Married	2	13.33	2	13.33	4	13.33
Types of substance use						
Ice	5	33.33	4	26.67	9	30.00
Amphetamines	2	13.33	2	13.33	4	13.33
Amphetamines and Ice	–	–	1	6.67	1	3.33
Cocaine	–	–	–	–	–	–
All	8	53.33	7	46.67	15	50.00
Not specified	–	–	1	6.67	1	3.33
Duration of substance use						
Less than 2 years	1	6.67	1	6.67	2	6.67
2 – 5 years	1	6.67	4	26.67	5	16.67
6 – 10 years	3	20.00	–	–	3	10.00
More than 10 years	10	66.67	10	66.67	20	66.67
Duration of rehabilitation						
Less than 1 year	8	53.33	11	73.33	19	63.33
1 – 2 years	5	33.33	3	20.00	8	26.67
3 – 5 years	1	6.67	1	6.67	2	6.67
More than 5 years	1	6.67	–	–	1	3.33
Physical illnesses						
No	13	86.67	13	86.67	26	86.67
Yes	2	13.33	2	13.33	4	13.33
Mental Illnesses						
No	15	100.00	14	93.33	29	96.67
Yes	–	–	1	6.67	1	3.33

#### 3.1.1 Efficacy of integrative cognitive neuropsychological program emphasizing brain response to enhance inhibitory control (ICNIC Intervention Program).

The inhibitory control of substance use among the recruited participants was assessed using a self-report measure and a cognitive performance task to evaluate the efficacy of the ICNIC intervention program. The BICI-SU was implemented to assess self-efficacy on behaviors associated with inhibitory control, while, the SSST was used to examine the cognitive performance of inhibitory control among these abusers during three periods: before ICNIC training (pre-training), after ICNIC training (post-training), and in a three-week follow-up after ICNIC training period.

#### 3.1.2 Analyzed results of behavioral inhibitory control inventory - substance use (BICI-SU)

An analysis of variance (ANOVA) was conducted to examine the BICI-SU scores across groups and time intervals. Before proceeding with further analyses, Mauchly’s Test of Sphericity was performed to assess the assumption of sphericity (**Table 2**). The results indicated a Mauchly’s W of 0.83, a Chi-Square value of 4.76, and a *p*-value of 0.092. Since the *p*-value is above the threshold of 0.05, the assumption of sphericity was not violated, suggesting that the variances of the differences between conditions are approximately equal and thus satisfying the condition of compound symmetry.

**Table 2:** Results of compound symmetry of behavioral inhibitory control inventory - substance use (BICI-SU).

Within subjects effect	*Mauchly’s W*	*Approx. Chi-Square*	*df*	*p*	*Epsilon*		
					*Greenhouse-Geisser*	*Huynh-Feldt*	*Lower-bound*
Interval	0.83	4.76	2	0.092	0.86	0.94	0.50

The researchers conducted a repeated-measures analysis of variance (ANOVA) on the mean inhibitory control scores at three time points—pre-ICNIC training, post-ICNIC training, and three-week follow-up training—to assess the main effects. **Table 3** demonstrates the results of two-way mixed-design ANOVA.

**Table 3:** Statistical summary of ANOVA: effects of groups and measurement intervals on inhibitory control.

Source of variation	*SS*	*df*	*MS*	*F*	*p*	*η^2^*
Between Subjects	68913.77	28	20068.74			
Group	18190.09	1	18190.09	9.68*	0.004	0.26
Error	50723.68	27	1878.66			
Within Subjects	73102.47	58	27161.72			
Interval	45554.79	2	22777.40	63.07*	0.000	0.70
Interval X Group	8046.38	2	4023.19	11.14*	0.000	0.29
Error (Interval)	19501.30	54	361.14			
Total	142016.24	86	47230.47			

*Significant Level at 0.05; n/s, No significance.

The interaction between intervals and groups yielded a sum of squares (*SS*) of 8046.38, with a mean square (*MS*) of 4023.19. The *F*-distribution value was 11.14, with a *p*-value of <0.001 and a partial eta-squared (*η^2^*) effect size of 0.29, indicating a moderate to large effect. These results suggest that the interaction between intervals and groups had a statistically significant effect on the mean scores of inhibitory control at the 0.05 level. This significant interaction implies that the effect of intervals on inhibitory control varied depending on groups.

Additionally, the analysis revealed that the measurement interval (Interval) had a sum of squares (*SS*) of 45,554.79, a mean of squares (*MS*) of 22,777.40, an *F*-value of 63.07, a *p*-value of <0.001, and an effect size (Cohen’s d) of 0.70, indicating a significant effect on the mean inhibitory control scores at the 0.05 level. Furthermore, the experimental method (Group) showed a sum of squares (*SS*) of 18,190.09, a mean of squares (*MS*) of 18,190.09, an *F*-value of 9.68, a *p*-value of 0.004, and an effect size (Cohen’s d) of 0.26. The results suggest that the experimental method (Group) showed a significant effect on the mean inhibitory control scores.

Comparing the mean behavioral inhibitory control scores of BICI-SU within the ICNIC groups reveals significant improvements of inhibitory control, as demonstrated in **Table 4**. Post-training scores (215.40 ± 18.79) were significantly higher than pre-training scores (188.73 ± 34.14) at the 0.05 level. Furthermore, three-week follow-up scores (265.53 ± 12.85) were significantly elevated compared to both pre-training and post-training scores at *p*-value < 0.05.

**Table 4:** Descriptive statistics and statistical comparison between groups and within groups of BICI-SU scores in three periods: pre-training, post-training, and 3-week follow-up.

Inhibitory control	Pre-training		Post-training		Follow-up	
	ICNIC group	Control group	ICNIC group	Control group	ICNIC group	Control group
1. Interference Control	102.87 (20.19)	102.33 (23.87)	120.73 (10.22)	101.80 (24.49)	147.13 (2.30)	117.21 (12.39)
1.1 Cognitive Inhibition	62.47 (12.97)	62.93 (13.81)	73.73 (6.42)	61.27 (14.61)	88.93 (1.28)	69.14 (7.50)
1.2 Selective Attention	40.40 (8.87)	39.40 (10.45)	47.00 (4.36)	40.53 (10.20)	58.20 (1.27)	48.07 (5.62)
2. Response Inhibition	85.87 (15.07)	84.07 (15.41)	94.67 (10.21)	85.00 (21.47)	118.40 (1.06)	98.00 (8.54)
2.1 Self-Control	41.20 (7.44)	39.80 (6.46)	45.53 (4.87)	41.33 (10.01)	59.00 (.76)	47.14 (5.56)
2.2 Discipline	44.67 (8.51)	44.27 (9.21)	49.13 (6.09)	43.67 (11.70)	59.40 (.74)	50.86 (4.64)
Inhibitory Control (Total)	188.73 (34.14)	186.40 (37.40)	215.40 (18.79)	186.80 (45.11)	265.53 (12.85)	215.21 (19.43)

Groups	Intervals of measurement			*P-value*	Pairwise comparison	
	Pre-training	Post-training	Follow-up			
ICNIC Group	188.73 (34.14)	215.40 (18.79)	265.53 (12.85)	0.000*	Post-Training > Pre-Training Follow-Up > Pre-Training Follow-Up > Post-Training	
Control Group	186.40 (37.40)	186.80 (45.11)	215.21 (19.43)	0.000*	Follow-Up > Pre-Training Follow-Up > Post-Training	
*p-*value	*0.788*	0.013*	0.000*			
Group Comparison	n/s	ICNIC Group > Control Group	ICNIC Group > Control Group			

*Significant Level at 0.05; n/s, No significance.

Regarding within-group comparison for control groups, the results showed no significant differences between pre-training scores (186.40 ± 37.40) and post-training scores (186.80 ± 45.11). However, the results showed significantly higher mean behavioral inhibitory control scores at the three-week follow-up (215.21 ± 19.43) compared to both their pre-training and post-training scores at *p*-value < 0.05.

When comparing the mean behavioral inhibitory control scores between the groups, the ICNIC group showed markedly higher scores than the control group in relation to both the post-training scores (215.40 ± 18.79 > 186.80 ± 45.11, *p* < 0.05) and at the three-week follow-up scores (265.53 ± 12.85 > 215.21 ± 19.43, *p* < 0.05). However, there were no significant differences noted between the pre-training scores of the ICNIC group (188.73 ± 34.14) and the control group (186.40 ± 37.40) with a *p*-value of 0.788, which was consistent with the equal baseline level of behavioral inhibitory control between groups. Thus, these results indicate greater self-reported efficacy among substance abusers in terms of their ability to exhibit behavioral inhibitory control after ICNIC training.

#### 3.1.3 Analysis of the results of cognitive performance task: stop signal substance task, both visual and auditory tasks

Performance-based tasks were conducted to assess inhibitory control among substance abusers. **Table 5** demonstrates the descriptive statistics of SSST scores across three time points: pre-training, post-training, and a three-week follow-up.

**Table 5:** Descriptive statistics of Stop Signal Substance Task (SSST): visual and auditory tasks of substance abusers (ICNIC group and non-training control group) in three periods: pre-training, post-training, and 3-week follow-up.

SSST		Pre-training		Post-training		Follow-Up	
		ICNIC group	Control group	ICNIC group	Control group	ICNIC group	Control group
Go Accuracy (%)	Visual	92.93 (7.47)	92.10 (5.10)	95.46 (3.66)	93.20 (5.06)	88.86 (24.12)	92.75 (3.18)
	Audio	91.86 (6.82)	88.55 (6.14)	94.28 (5.01)	90.55 (5.97)	89.14 (24.76)	92.85 (4.17)
Go Reaction Time (ms)	Visual	346.64 (34.79)	335.21 (24.36)	342.08 (18.08)	319.91 (19.91)	362.59 (30.03)	339.97 (22.90)
	Audio	333.59 (21.20)	323.93 (20.16)	331.96 (17.74)	312.56 (14.17)	358.70 (31.96)	336.98 (25.50)
Stop Accuracy (%)	Visual	91.43 (26.43)	97.80 (3.19)	99.00 (1.88)	98.20 (2.20)	99.00 (1.71)	98.80 (1.93)
	Audio	89.28 (25.98)	95.20 (5.75)	98.43 (3.25)	96.40 (4.70)	95.43 (4.11)	91.80 (6.29)
Stop Signal Delay (ms)	Visual	226.13 (26.14)	217.93 (25.31)	230.54 (16.72)	218.29 (26.87)	231.55 (14.13)	228.59 (14.91)
	Audio	201.86 (68.45)	200.42 (65.28)	229.74 (48.56)	218.81 (39.17)	200.48 (55.96)	175.51 (77.58)
Stop Signal Reaction Time: SSRT (ms)	Visual	120.52 (38.85)	117.28 (29.46)	111.54 (23.09)	101.62 (29.72)	131.04 (34.98)	111.39 (34.24)
	Audio	131.73 (73.67)	123.52 (70.60)	102.23 (50.96)	93.74 (36.90)	158.22 (75.10)	161.47 (75.36)

From the stop-signal reaction time (SSRT) descriptive data, shorter SSRT reflects stronger inhibitory control. Our results showed a trend of improved inhibitory control only at the post-training periods in both ICNIC group (SSRT; visual: 111.54 ± 23.09 ms, auditory: 102.23 ± 50.96 ms) and non-training control group (SSRT; visual: 101.62 ± 29.72 ms, auditory: 93.74 ± 36.90 ms) when compared within groups. No trends were evident at pre-training or follow-up. However, none of these differences were considered to be statistically significant.

A slower ‘Go’ reaction time (Go RT) may indicate a more cautious or deliberate response strategy. Our results showed a trend toward slower Go RT among ICNIC group (Go RT; visual: 342.08 ± 18.08 ms, auditory: 331.96 ± 17.74 ms) compared to the non-training control group (Go RT; visual: 319.91 ± 19.91 ms, auditory: 312.56 ± 14.17 ms) during the post-training period. This pattern persisted during the three-week follow-up period, where the ICNIC group exhibited slower Go RT (Go RT; visual: 362.59 ± 30.03 ms, auditory: 358.70 ± 31.96 ms) as compared to the pre-training period (Go RT; visual: 346.64 ± 34.79 ms, auditory: 333.59 ± 21.20 ms).

The higher Stop-trial accuracy is typically interpreted as evidence of enhanced inhibitory control. The ICNIC group showed a trend toward improved Stop-trial accuracy during both the post-training (%St ACC; visual: 99.00 ± 1.88%, auditory: 98.43 ± 3.25%) and the three-week follow-up period assessments (%St ACC; visual: 99.00 ± 1.71%, auditory: 95.43 ± 4.11%) as compared to pre-training (%St ACC; visual: 91.43 ± 26.43%, auditory: 89.28 ± 25.98%). Nonetheless, these trends did not constitute statistical significance.

Although Go-trial accuracy varied across the three time points, no consistent improvement was observed. Overall, there were no statistically significant variations within-subject changes from pre- to post-training or in the follow-up period in terms of SSRT, Stop-trial accuracy, or Go-trial accuracy.

An ANOVA was conducted to examine SSST scores across groups and time intervals. Before proceeding with further analyses, Mauchly’s Test of Sphericity was performed to assess the assumption of sphericity (**Table 6**). The results indicated that the variables visual_SSD, visual_SSRT, audio_RT, audio_SSD, and audio_SSRT met the assumption of compound symmetry, indicating no violation of sphericity. However, for visual_%ACC, visual_RT, visual_%ACC-Stop, audio_%ACC, and audio_%ACC-Stop, sphericity was violated; thus, the Greenhouse–Geisser correction was applied.

**Table 6:** Results of compound symmetry of Stop Signal Substance Task (SSST): visual and auditory tasks.

Within subjects effect	Mauchly’s W	Approx. Chi-Square	df	p	Epsilon		
					Greenhouse-Geisser	Huynh-Feldt	Lower-bound
Visual_%ACC	0.32	23.86	2	0.000	0.60	0.64	0.50
Visual_RT	0.59	11.00	2	0.004	0.71	0.78	0.50
Visual_%ACC-Stop	0.04	69.37	2	0.000	0.51	0.54	0.50
Visual_SSD	0.89	2.43	2	0.296	0.90	1.00	0.50
Visual_SSRT	0.88	2.64	2	0.297	0.89	1.00	0.50
Audio_%ACC	0.13	42.99	2	0.000	0.54	0.57	0.50
Audio_RT	0.76	5.75	2	0.056	0.81	0.90	0.50
Audio_%ACC-Stop	0.14	42.02	2	0.000	0.54	0.57	0.50
Audio_SSD	0.85	3.31	2	0.191	0.87	0.99	0.50
Audio_SSRT	0.88	2.71	2	0.258	0.89	1.00	0.50

**Table 7** shows an inferential statistical analysis of SSST visual scores for the ICNIC training group and the non-training control group across three time points: pre-training, post-training, and the three-week follow-up. Our results demonstrate that the ICNIC training group (Go RT: 342.08 ± 18.08 ms) exhibited a significantly slower Go RT as compared to the non-training control group (Go RT: 319.91 ± 19.91 ms) during post-training period with statistical significance (*p*-value = 0.007). Furthermore, the ICNIC training group (Go RT: 362.59 ± 30.03 ms) continued to show a statistically significant slower Go RT score than the non-training control group (Go RT: 339.97 ± 22.90 ms) during the three-week follow-up period (*p*-value = 0.047). Meanwhile, the non-training control group demonstrated a significantly faster Go RT at post-training period (Go RT: 319.91 ± 19.91 ms) than the three-week follow-up period (Go RT: 339.97 ± 22.90 ms) with a *p*-value = 0.016. This suggests that, the ICNIC group exhibited a more cautious and better response strategy to optimize inhibitory control. This is often interpreted as a form of proactive control or a compensatory adjustment aimed at enhancing inhibitory task performance.

**Table 7:** Statistical comparison between groups and within groups of Stop Signal Substance Task (SSST): visual tasks.

Variables	Groups	SSST (Visual)			P-value	Pairwise comparison
		Pre-training	Post-training	Follow-up		
Go Accuracy (%)	ICNIC Group	92.93	95.46	88.86	0.430	n/s
	Control Group	92.10	93.20	92.75	0.808	n/s
	*p-value*	0.837	0.666	0.772		
	Pairwise Comparison					
Go Reaction Time (ms)	ICNIC Group	346.64	342.08	362.59	0.158	n/s
	Control Group	335.21	319.91	339.97	0.016*	Follow-Up > Post-Training
	*p-value*	0.349	0.007*	0.047*		
	Pairwise Comparison	n/s	ICNIC Group > Control Group	ICNIC Group > Control Group		
Stop Accuracy (%)	ICNIC Group	91.43	99.00	99.00	0.294	n/s
	Control Group	97.80	98.20	98.80	0.407	n/s
	*p-value*	0.431	0.860	0.228		
	Pairwise Comparison	n/s	n/s	n/s		
Stop Signal Delay (ms)	ICNIC Group	226.13	230.54	231.55	0.575	n/s
	Control Group	217.93	218.29	228.59	0.296	n/s
	*p-value*	0.654	0.556	0.225		
	Pairwise Comparison	n/s	n/s	n/s		
Stop Signal Reaction Time: SSRT (ms)	ICNIC Group	120.52	111.54	131.04	0.207	n/s
	Control Group	117.28	101.62	111.39	0.210	n/s
	*p-value*	0.699	0.192	0.535		
	Pairwise Comparison	n/s	n/s	n/s		

*Significant Level at 0.05; n/s, No significance.

However, there were no significant differences in SSRTs between the ICNIC training and non-training control groups across the three time points. Likewise, other SST outcome measures did not show any significant differences between groups.

**Table 8** shows the inferential statistical analysis of SSST auditory scores among ICNIC training group and the non-training control group at three time points: pre-training, post-training, and in the three-week follow-up. Our results demonstrate that the ICNIC training group (Go RT: 331.96 ± 17.74 ms) exhibited significantly slower Go RT compared to the non-training control group (Go RT: 312.56 ± 14.17 ms) during post-training period, with statistically significant *p*-value of 0.009). Furthermore, the ICNIC training group exhibited a markedly slower Go RT during the three-week follow-up period (Go RT: 358.70 ± 31.96 ms) when compared to pre-training period (Go RT: 333.59 ± 21.20 ms) and post-training period (Go RT: 331.96 ± 17.74 ms) with a statistically significant *p*-value of 0.001), suggesting an increase in response caution over time. Meanwhile, in the non-training control group, Go RTs during the three-week follow-up period (Go RT: 336.98 ± 25.50 ms) and the pre-training period (Go RT: 323.93 ± 20.16 ms) were significantly slower than the post-training period (Go RT: 312.56 ± 14.17 ms), with a *p*-value of 0.001.

**Table 8:** Statistical comparison between groups and within groups of Stop Signal Substance Task (SSST): audio tasks.

Variables	Groups	SSST (Audio)			P-value	Pairwise comparison
		Pre-training	Post-training	Follow-up		
Go Accuracy (%)	ICNIC Group	91.86	94.28	89.14	0.542	n/s
	Control Group	88.55	90.55	92.85	0.041	n/s
	*p-value*	0.235	0.110	0.646		
	Pairwise Comparison					
Go Reaction Time (ms)	ICNIC Group	333.59	331.96	358.70	0.001*	Follow-Up > Pre-Training Follow-Up > Post-Training
	Control Group	323.93	312.56	336.98	0.001*	Post-Training < Pre-Training Follow-Up > Post-Training
	*p-value*	0.273	0.009*	0.089		
	Pairwise Comparison	n/s	ICNIC Group > Control Group	n/s		
Stop Accuracy (%)	ICNIC Group	89.28	98.43	95.43	0.284	n/s
	Control Group	95.20	96.40	91.80	0.063	n/s
	*p-value*	0.489	0.223	0.101		
	Pairwise Comparison	n/s	n/s	n/s		
Stop Signal Delay (ms)	ICNIC Group	201.86	229.74	200.48	0.266	n/s
	Control Group	200.42	218.81	175.51	0.183	n/s
	*p-value*	0.959	0.563	0.368		
	Pairwise Comparison	n/s	n/s	n/s		
Stop Signal Reaction Time: SSRT (ms)	ICNIC Group	131.73	102.23	158.22	0.048*	Follow-Up > Post-Training
	Control Group	123.52	93.74	161.47	0.035*	Follow-Up > Post-Training
	*p-value*	0.787	0.658	0.918		
	Pairwise Comparison	n/s	n/s	n/s		

*Significant Level at 0.05; n/s, No significance.

Statistical analysis of the SSST auditory scores demonstrated that the ICNIC group demonstrated a trend toward improved inhibitory control with a shorter SSRT during the post-training period (SSRT: 102.23 ± 50.96 ms) compared to the pre-training period (SSRT: 131.73 ± 73.67 ms), even though this difference was not statistically significant. However, SSRTs significantly increased at the three-week follow-up period (SSRT: 158.22 ± 75.10 ms) as compared to the post-training period (*p*-value = 0.048), indicating a decline in inhibitory control performance after training cessation. This could imply a potential immediate effect of the ICNIC program on inhibitory control among substance abusers during post-training period. The non-training control group exhibited a similar pattern, with SSRTs showing a non-significant decrease from the pre-training period (SSRT: 123.52 ± 70.60 ms) to the post-training period (SSRT: 93.74 ± 36.90 ms), followed by a significant increase at the three-week follow-up (SSRT: 161.47 ± 75.36 ms) as compared to the post-training period with a *p*-value of 0.035).

In regard to between-group comparison, there were no significant differences noted in SSRTs between the ICNIC training and non-training control groups across the three time points. Likewise, no other SST outcome measures indicated any significant differences between groups.

#### 3.1.4 Analysis of the results of participants’ satisfaction and feedback questionnaire and interviews: quantitative and qualitative data

The researchers collected the feedback data from substance abusers on the ICNIC program using a 23-item questionnaire with a Cronbach’s alpha coefficient of 0.95 and discrimination values between 0.29 - 0.88. Based on responses using a 5-point Likert scale, our results revealed that ICNIC-trained substance abusers exhibited a high level of satisfaction with the ICNIC intervention program (3.98 ± 0.51), as shown in **Table 9**.

**Table 9:** Descriptive statistics of ICNIC intervention program satisfaction and feedback.

Statements	Mean	S.D.
1. You are relaxed and enjoying the training.	3.80	0.63
2. The ICNIC program help you create an atmosphere and build good relationships.	4.13	0.48
3. The ICNIC program help you gain knowledge about the effects of drugs on the brain and enhancing brain resilience.	4.20	0.63
4. The ICNIC program help you develop skills for brain rehabilitation.	4.27	0.66
5. The ICNIC program help you gain knowledge about inhibitory control.	4.07	0.66
6. The ICNIC program help you develop skills in enhancing inhibitory control.	4.00	0.71
7. The ICNIC program help you apply knowledge and skills to enhance inhibitory control.	3.73	0.75
8. The ICNIC program help you identify the values and goals of enhancing inhibitory control.	3.87	0.78
9. The ICNIC program help in gaining knowledge and skills to accept or face life situations that were once avoided or escaped.	3.80	0.73
10. The ICNIC program provide you with a way to apply acceptance skills in daily life.	4.07	0.56
11. The ICNIC program help you stay in the present moment.	4.13	0.70
12. The ICNIC program help you detect past experiences related to substance use.	3.93	0.75
13. The ICNIC program help you identify the triggers (images, sounds, or feelings) that are causes of substance addiction.	3.87	0.78
14. The ICNIC program provide you with a way to apply knowledge and skills for staying present to enhance self-control in daily life.	3.87	0.70
15. The ICNIC program help you develop a way to apply knowledge and skills in separating thoughts to enhance self-control in daily life.	3.93	0.66
16. The ICNIC program help you apply knowledge and skills in being aware of changes in daily life.	4.07	0.66
17. The ICNIC program help you identify practices for enhancing self-control.	3.93	0.66
18. The ICNIC program help you identify internal and external obstacles to enhancing self-control, as well as strategies for preventing these obstacles.	3.80	0.63
19. The ICNIC program help you improve and adjust the action plan for enhancing self-control after it has been implemented in daily life.	3.73	0.66
20. The ICNIC program allow you to exchange experiences regarding challenges and obstacles in enhancing inhibitory control.	3.80	0.73
21. The ICNIC program help you develop brain control and inhibition.	3.87	0.70
22. The researcher uses simple language and presents the activities in a step-by-step manner.	4.33	0.58
23. Overall satisfaction with the course.	4.33	0.76
Total	3.98	0.51

The ICNIC-trained substance abusers provided additional feedbacks on the program, which could be summarized as following: 1) they would like the ICNIC program to be implemented every year for monitoring brain functions, 2) the duration of program was too short because some participants had limited time, 3) they wanted more action-oriented activities rather than thought-provoking methods, 4) the program activities were well-developed, but the time was insufficient, 5) the program activities helped to increase consciousness when experiencing cravings for drugs or other stimuli, 6) it would be beneficial to have more activities that can be practiced, 7) they suggested adding informative video clips about the brain, 8) they expressed that the program activities were beneficial for them as well as for other drug abusers, 9) they wanted more academic lectures or workshops with experts for a large group of members, and 9) they wanted more media, video clips, slides, etc. to be included.

Furthermore, the activities of ICNIC program were diverse and provided opportunities to establish values and goals, as well as improve personal practices. The ICNIC-trained substance abusers offered their thoughts toward inhibitory control during program activities as following:

Mr. A: *“I have applied inhibitory control skills in breathing exercises. The obstacles are distracting noises and interactions with people around me.” Regarding the Tower of Hanoi game, “I couldn’t manage my time.” As for the practice of accepting words that one dislikes, “I have adjusted my choices by practicing letting go of thoughts, being aware of changes, practicing breathing for five minutes, and body scanning for five minutes.”*

Mr. B: *“I have refocused on my set goals and practiced flexible thinking. The activities helped increase my mindfulness when experiencing cravings for drugs and other stimuli.”*

Mr. C: *“I have improved my choices by playing* sp*orts, practicing meditation, and breathing. Playing* sp*orts makes me feel good and relaxes my brain. Meditation helps me stay in the present moment more. Breathing exercises help me to be more mindful before doing anything.”*

Mr. D: *“I have become aware of my own and others’ actions and obstacles, such as my own lack of concentration, laziness, boredom, low patience, and sleep time.”*

Mr. E: *“I have learned about the outcomes of my choices, my goals, and how to analyze and persevere with the things I choose. I have realized my own determination towards achieving the things I choose.”*

## 4 Discussion

This original research study was conducted to examine the efficacy of a cognitive rehabilitation program, the ICNIC intervention program, in order to enhance inhibitory control in the context of substance use. Quantitative and qualitative data were collected using BICI-SU, SSST, a feedback questionnaire, and reflection interviews. The substance abusers were recruited as outpatients from alternative drug rehabilitation centers, and they were predominantly psychostimulant users with over ten years of drug use history and less than one to two years of rehabilitation. Using a self-report measure (BICI-SU) our results demonstrated these substance abusers reported greater self-efficacy in their ability to engage in behaviors associated with inhibitory control following the ICNIC training.

When we compared training and non-training substance abusers, the BICI-SU data revealed that the ICNIC intervention program could increase behavioral inhibitory control of substance use among these abusers, indicating its potential efficacy in behavioral modification regarding the inhibitory control of substance use, although further studies are needed to elucidate these findings. When assessed using the cognitive performance task SSST, no improvements in inhibitory control were observed, as indicated by the ‘Go’ or ‘Stop’ trial accuracy and SSRT. Additionally, the lack of improved accuracy coincided with slower responses to ‘Go’ stimuli in the ICNIC group when compared to the non-training control group. The slower response of ‘Go’ stimuli could indicate a more cautious and better response strategy for optimizing inhibitory control.

Alternatively, the observed group differences could be driven by faster responses in the non-training control group rather than slower responses in the ICNIC group. Therefore, further studies are needed to validate and interpret these findings comprehensively. However, these substance users nonetheless demonstrated positive responses and reported satisfaction with the ICNIC intervention program. They reflected that the program activities and contents were very helpful in controlling thoughts and behaviors associated with the inhibitory control of substance use.

Inhibitory control deficits are behavioral and cognitive problems among individuals with diverse forms of addiction, such as substance abuse, gambling, internet usage, video game, sexual activities, and shopping (25, 57). The impairment of inhibitory control among substance abusers is the detrimental consequence of prolonged drug use. Subsequently, dysfunctional inhibition is identified as a primary risk factor for relapse and drug-seeking behaviors (26, 58).

Extending from previous research studies, a variety of self-report measures and cognitive performance tasks have been used to assess inhibitory control, impulsivity, and related cognitive constructs among individuals with SUDs. For example, Barratt Impulsiveness Scale-11 (BIS-11) has been utilized to assess impulsivity among cocaine-dependent and methamphetamine-dependent patients to predict the treatment completion (59). UPPS-P Impulsive Behavior Scale has also been used to measure impulsivity among SUD in-treatment patients to examine the effectiveness of Mindfulness-Based Relapse Prevention (MBRP). UPPS-P Scale was implemented along with a cognitive performance task, the Stroop Word Color Test, to assess impulsivity and inhibitory control, respectively. However, this previous study showed no significant effect of MBRP on impulsivity dimensions and inhibitory control in patients SUDs in the therapeutic community (60).

On the contrary, our study demonstrated that the incorporation of the ICNIC intervention program with regular rehabilitative programs at the treatment centers can provide the additional therapeutic effect on behavioral inhibitory control, as assessed by the self-report measure BICI-SU. The substance abusers reported greater self-efficacy in engaging in behaviors related to inhibitory control over substance use. Further, our data arising from a cognitive performance task, SSST, did not show any improvement in inhibitory control, even though some parts of the SSST data demonstrated a potential link to an improved or more cautious response strategy that can be used to optimize inhibitory control. Thus, in this study, the self-report measure and the standardized laboratory tasks of inhibitory control did not correlate in evaluating the efficacy of the ICNIC intervention program. Consistent with previous studies, meta-analytic evidence has shown that self-report measures and task-based assessments of cognitive control do not overlap and might capture different psychological processes or constructs (61).

A further report has also indicated a substantial lack of correlation between self-report measures and performance-based tasks, focusing on different aspects of the theoretical construct of self-control (62). The distinction among these two measurements can be explained as follows: 1) self-report measures assess typical or habitual behaviors while tasks assess maximum or optimal performance under certain condition, 2) self-report measures reflect general behavioral patterns while tasks capture momentary and state-level responses, and 3) self-report measures assess broad and cross-domain inhibition while tasks assess narrow and domain-specific control (62). Thus, in the context of cognitive assessments, task-based measures and self-report measures may best be viewed as complementary, yet largely distinct, perspectives on cognitive control (63). In our study, incorporating both types of measurements has provided a more comprehensive assessment of inhibitory control.

In addition, previous research findings have indicated that standardized residential substance abuse treatment could exert therapeutic effects on inhibitory control over the course of treatment, as measured by the UPPS-P Impulsive Behavior Scale and a computer-administered stop-signal task, STOP-IT (64). Consistent with our results in this present study, non-training substance abusers who received only regular rehabilitative program at drug treatment centers also exhibited high levels of inhibitory control during the three-week follow up period, as assessed by both BICI-SU and SSST. Accordingly, initial signs of therapeutic outcomes in inhibitory control were observed in the regular rehabilitative program. Furthermore, the incorporation of the ICNIC program as an additional cognitive rehabilitation approach, with a direct emphasis on inhibitory control, appears to provide therapeutic benefits in drug treatment, preventing relapse and drug-seeking behaviors.

The ICNIC program was a researcher-developed, integrative cognitive neuropsychological program which aimed to foster inhibitory control as a therapeutic outcome in the context of drug addiction. This program was designed based on the fundamental principles of neuroscience, cognitive psychology, and Acceptance and Commitment Therapy (ACT). It has been positively evaluated by experts in the fields.

Our results demonstrated the beneficial rehabilitative effects of ICNIC program in terms of fostering behavioral inhibitory control and more cautious response strategies as long-term therapeutic outcomes. Several lines of research evidence have indicated that some common characteristics of patients with SUDs undergoing drug treatment and rehabilitation include stress, cue-induced cravings, and loss of cognitive control. The currently applied therapeutic approaches have focused on cognitive-behavioral strategies, aiming to modify behavior that ultimately results in reduced consumption or abstinence in patients with SUDs (65).

Previous research findings have also shown the positive effects of cognitive training and cognitive rehabilitation in the context of drug addiction (30). Cognitive deficits are common clinical manifestations in patients with SUDs (15, 16). Moreover, cognitive training exerts a beneficial effect upon the improvement of cognitive functions, including attention, memory, executive functions, abstract reasoning, problem solving, and processing speed among SUD patients, subsequently reducing craving symptoms and drug relapse (30).

Cognitive rehabilitation or remediation techniques primarily use cognitive-behavioral approaches in the treatment of addictive behaviors by incorporating meta-cognitive training and strategy learning that emphasizes goal-directed behaviors, decision-making (16), and cognitive control (35). In addition, these cognitive rehabilitative approaches aim to enhance strategies for coping with stressful conditions and cue-induced craving, as well as targeting erroneous learning mechanisms (65). Such techniques include standard CBTs, the cognitive inhibition of craving, neurofeedback training on addiction, neuromodulation, emotional regulation, mindfulness-based interventions, motivational interventions, cognitive bias modification, reconsolidation-based interventions, and virtual-reality-based cue exposure therapy, as well as pharmacological augmentation strategies (35, 65). Thus, both cognitive function and cognitive rehabilitation focus on brain regions associated with higher cognitive functions, including the PFC and amygdala (65, 66).

Correspondingly, ACT interventions have been shown to primarily promote psychological flexibility and directly improve cognitive function in certain domains, including executive functions, attention, memory, and subjective cognitive functions (67). Furthermore, ACT interventions have been applied in drug treatment settings (36), where they have been shown to improve psychological flexibility and alleviate psychological symptoms such as depression, anxiety, and shame among patients with SUDs (37, 41–43). Other research evidence has demonstrated that ACT can effectively reduce impulsivity (44) and s indicates a trend in positive improvement on executive functions, including inhibitory control, task monitoring, and emotional control (45).

In the present study, the ICNIC program applied the concepts of ACT along with neurocognitive stimulation techniques as an additional cognitive rehabilitation program to enhance patients’ therapeutic outcomes at alternative drug rehabilitation centers. The program activities targeted higher cognitive processes, such as memory, reasoning, decision-making, cognitive flexibility, goal-directed persistence, and inhibitory control. Our results in this study could confirm the potential effectiveness of the ICNIC program in enhancing behavioral inhibitory controls in the context of substance use, fostering inhibitory control as a long-term therapeutic outcome.

Supporting the findings in this study, previous clinical and preclinical research studies have also shown that cognitive training and cognitive stimulation through new learning experiences can improve cognitive impairment and drug-induced symptoms by promoting adaptive neuroplastic changes (68). Finally, our ICNIC-training substance abusers indicated high levels of satisfaction towards program activities and gave positive feedback in controlling thoughts and behaviors associated with inhibitory control of substance use.

Similarly, adults and adolescents who received ACT-based interventions demonstrated moderate to high levels of satisfaction (69, 70). Universal ACT web-based intervention has been used to promote well-being and prevent mental health problems among adolescents. Research results demonstrated that adolescents who spend more time performing intervention activities exhibited higher levels of satisfaction, while, adolescents with less time usage on intervention exhibited lower levels of satisfaction (69). In addition, adults with eating disorders stated that they were somewhat satisfied to very satisfied with the open ACT group and that the treatment content was helpful (70).

## 5 Limitations and future research

This research study examined the beneficial effects of a supplementary ICNIC intervention program appended to regular drug treatment for promoting inhibitory control over substance use. However, this study contains some limitations. First, the small sample size might lead to non-generalizable results in terms of expressing the efficacy of ICNIC intervention programs. Second, the cross-sectional study design may have served to reduce the statistical impact of efficacy in ICNIC intervention programs. Longitudinal studies might provide a better understanding of the efficacy of an ICNIC program on inhibitory control in terms of long-term therapeutic outcomes and adaptive neuroplastic changes. Third, our use of a self-reporting measure, BICI-SU, could undermine the reliability of study results. Finally, the cognitive performance task, SSST, did not indicate any correlation with the self-report measure.

Future research studies might therefore consider incorporating additional cognitive performance tasks to measure the efficacy of ICNIC intervention programs on cognitive functions, particularly executive functions. An increase of the sample size to elevate the reliability of the research results should also be considered.

## 6 Conclusion

The ICNIC intervention program revealed an improvement in self-efficacy in inhibitory control over substance use and may therefore be associated with more cautious behavioral response strategies, as reflected through inhibitory control tasks having slower response times. However, further research is needed to confirm and expand upon these findings.

The ICNIC intervention program, when used as a supplementary cognitive rehabilitation program, appears to enhance behaviors linked to inhibitory control. The program’s activities appear to contribute to cognitive stimulation which increases inhibitory control and promotes behavioral changes. Hence, we suggest that the program might be implemented as an additional intervention over and above regular rehabilitative programs in order to increase the effectiveness of drug treatment strategies and thereby contribute to more positive therapeutic outcomes.

## Data Availability

The raw data supporting the conclusions of this article will be made available by the authors, without undue reservation.
